# Irinotecan plus leucovorin-modulated 5-fluorouracil I.V. bolus every other week may be a suitable therapeutic option also for elderly patients with metastatic colorectal carcinoma

**DOI:** 10.1038/sj.bjc.6601214

**Published:** 2003-09-09

**Authors:** P Comella, A Farris, V Lorusso, S Palmeri, L Maiorino, L De Lucia, F Buzzi, S Mancarella, F De Vita, A Gambardella

**Affiliations:** 1 Division of Medical Oncology, National Tumour Institute, Via M. Semmola, 80131 Naples, Italy; 2 Chair of Medical Oncology, University School of Medicine, Viale S. Pietro 8, 07100 Sassari, Italy; 3 Department of Medical Oncology, Oncology Institute, Via G. Amendola 209, 70126 Bari, Italy; 4 Chair of Medical Oncology, University School of Medicine, P.za delle Cliniche 2, 90127 Palermo, Italy; 5 Medical Oncology, San Gennaro Hospital, Via S. Gennaro dei Poveri 5, 80131 Naples, Italy; 6 Medical Oncology, City Hospital, Via G. Tescione 81, 81100 Caserta, Italy; 7 Medical Oncology, City Hospital, Via T. Joannuccio, 05100 Terni, Italy; 8 Medical Oncology, City Hospital, Via Taranto, 73021 Campi Salentino (Lecce), Italy; 9 Chair of Medical Oncology, Second University School of Medicine, Via S. Pansini, 80131 Naples, Italy; 10 Chair of Geriatrics, Second University School of Medicine, Via S. Pansini, 80131 Naples, Italy

**Keywords:** colorectal carcinoma, elderly patients, combination chemotherapy, Irinotecan, 5-fluorouracil

## Abstract

The aim of this study was to assess the safety and efficacy of biweekly irinotecan plus leucovorin-modulated 5-fluorouracil i.v. bolus in metastatic colorectal carcinoma according to the age of patients. For this purpose, we have analysed 108 patients randomly allocated to receive irinotecan 200 mg m^−2^ i.v. (1-h infusion) on day 1, and L-leucovorin 250 mg m^−2^ i.v. (1-h infusion) plus 5-fluorouracil 850 mg m^−2^ i.v. bolus on day 2 every 2 weeks (IRIFAFU) in our previous SICOG 9801 trial. According to age, patients were retrospectively divided into three groups: younger (⩽54 years, *n*=37), middle-aged (55–69 years, *n*=64), and elderly (⩾70 years, *n*=17). Apart from gender, pretreatment characteristics were well balanced across the three groups. WHO grade ⩾3 neutropenia and diarrhoea affected on the whole 46 and 16 patients, respectively, without any significant difference according to age-grouping. Patients aged ⩽54 years stayed on therapy for a longer time (median 24 *vs* 14–15 weeks), and received more cycles (median 9 *vs* 7), than the older ones. Only one patient in the young group withdrew consent to therapy as opposed to four patients each in the aged and elderly one. Response rate was 38% for younger patients, 34% for aged, and 35% for the elderly ones. Median time to progression was 7.4, 8.0, and 5.3 months, and median survival time was 13.4, 15.3, and 13.9 months, respectively. We conclude that IRIFAFU given every other week may represent a suitable therapeutic option also for elderly patients with metastatic colorectal carcinoma.

Colorectal carcinoma is among the most common cancers in western countries. In Italy, about 40 new cases per 100 000 males, and 20 new cases per 100 000 females are diagnosed each year (Zanetti *et al*, 1997). Most of these cases are discovered in patients aged 65 years or more.

In recent years, the postsurgical administration of 5-fluorouracil (FU)-based chemotherapy in high-risk (Dukes' stage C) colon cancer patients has been proven to reduce the recurrence and death rate (Wolmark *et al*, 1999). On the contrary, there is still no agreement about the absolute benefit that could be obtained in patients with more limited (Dukes' stage B) extension (International Multicenter Pooled Analysis of B2 Colon Cancer Trials, 1999; Mamounas *et al*, 1999). Anyway, recently published retrospective studies and meta-analyses have reported no interaction between age of patients and effect of adjuvant chemotherapy, suggesting that elderly patients should also be offered such treatments (Sargent *et al*, 2001a; Sundararajan *et al*, 2002).

Until recently, the usual management for the recurrent or metastatic disease included leucovorin (LV)-modulated FU chemotherapy (LV-FU), given in a 5-day monthly or in a once-a-week schedule (Buroker *et al*, 1994). These two regimens produced equivalent results, and a meta-analysis has shown that this palliative treatment was associated with a 35% reduction in the risk of death as compared with supportive care alone, which translated in an improvement in median survival of 3.7 months (Colorectal Cancer Collaborative Group, 2000). Also in this case, no age-related difference was found as to the effectiveness of chemotherapy (Chiara *et al*, 1998; Popescu *et al*, 1999).

Despite this observation, the number of elderly patients receiving palliative chemotherapy for colorectal carcinoma is still limited (Hutchins *et al*, 1999). Indeed, there is still a diffuse concern about the compliance and tolerability of chemotherapy in such patients (Daniele *et al*, 1999). On the other hand, some investigators have already stressed that performance status, and associated morbidities, more than age of patients, may adversely affect their outcome (Extermann *et al*, 1998; Yancik *et al*, 2001).

Recently, novel drugs such as irinotecan or oxaliplatin have shown activity in this disease when used either alone or in combination with FU. Addition of irinotecan to LV-FU has been proven to increase significantly the response rate, the time to progression, and the median survival of patients, in comparison with LV-FU alone (Douillard *et al*, 2000; Saltz *et al*, 2000). Also, oxaliplatin combined with LV-FU has demonstrated to increase significantly the response rate and the time to progression, but not the survival, in comparison with the same regimen without oxaliplatin (de Gramont *et al*, 2000). Therefore, irinotecan plus LV and FU is now considered the gold standard of treatment for metastatic patients. However, because no prospective analysis has been carried out on the tolerability of these new combinations in aged people, a special care has been recommended, in consideration of the early and unpredictable adverse events that may occur in older individuals (Rothemberg *et al*, 2001; Sargent *et al*, 2001b).

In recent years, the Southern Italy Cooperative Oncology Group (SICOG) has devised an original biweekly regimen, including irinotecan plus LV-modulated FU given as i.v. bolus (IRIFAFU) for metastatic colon cancer patients (Comella *et al*, 2000). The IRIFAFU combination has been compared in a randomised multicentre study (SICOG trial 9801) with FU modulated by methotrexate and LV. In that trial, the IRIFAFU regimen produced a greater response rate, and a longer time to progression, than the control treatment (Comella *et al*, 2002). Here we report the results of a retrospective analysis we have carried out on the patients randomly allocated to receive the combination regimen, with the aim of having a deeper insight on the interaction between age of patients, their tolerability to chemotherapy, and outcome.

## PATIENTS AND METHODS

### Patient population

Patients affected by metastatic colorectal cancer, enrolled into the SICOG trial 9801, and randomly allocated to receive the IRIFAFU regimen (experimental arm), were the object of this retrospective analysis. Briefly, eligibility criteria for SICOG trial 9801 were: histologically proven adenocarcinoma of the colon or rectum; presence of bidimensionally measurable lesion(s); age ⩾18 years; performance status ⩽2 of the Eastern Cooperative Oncology Group (ECOG) scale; adequate liver, renal, and bone marrow reserve. Patients previously treated with FU-based adjuvant chemotherapy were also included, provided that at least 6 months had elapsed from treatment discontinuation.

### Treatment

Patients in the experimental arm received the IRIFAFU regimen: irinotecan 200 mg m^−2^ given i.v. over 1-h on day 1, and L-leucovorin 250 mg m^−2^ given as 1-h i.v. infusion, followed by FU 850 mg m^−2^ as i.v. bolus, on day 2. Cycles were repeated every other week until progression, or for a maximum of 6 months. Attending physicians were required to specify the reason for an earlier treatment discontinuation. Disease status was checked every 2 months, and classified according to the World Health Organization (WHO) criteria (Miller *et al*, 1981). Adverse events of each treatment cycle were scored according to WHO scale (Miller *et al*, 1981), and the worst grade for each patient during treatment was recorded. Haematological toxicity was checked weekly. The actual dose intensity of cytotoxic drugs over 4 and 8 cycles (DI_4_ and DI_8_, respectively) was calculated as previously reported. Patients were followed every 2 months for assessing tumour progression and survival, and the cause of death (disease- or treatment-related, or due to other reasons) was recorded.

### Analysis of safety and activity

According to their age, patients were divided into three groups: younger (⩽54 years), middle-aged (55–69 years), and elderly (⩾70 years). Differences in distribution of pretreatment characteristics among the three groups were assessed by the *χ*^2^ test. Comparison of treatment in the three age groups (i.e., number of cycles, duration of therapy, and dose intensities) was made by the Kruskal–Wallis test. The difference in reasons for going off-study was assessed for significance by the Pearson *χ*^2^-test. For exploring the interaction between pretreatment characteristics and treatment activity, patients achieving a complete or partial response were classified as ‘responders’, while patients showing a stable or progressive disease, as well as those not assessed for response, were classified as ‘failures’ according to intent-to-treat analysis. The following characteristics were explored for correlation with activity: age, sex, primary site, previous radical surgery, previous adjuvant chemotherapy, time to occurrence of metastatic disease, performance status, presence of symptoms, significant loss of body weight, number of disease sites, and basal carcinoembryonic antigen (CEA) serum level. For statistical analysis, the following variables were coded as dichotomous: primary site (colon or rectum), performance status (0 or ⩾1), timing of metastasis (synchronous or metachronous), number of disease sites (1 or ⩾2), and CEA basal value (<100 or ⩾100 ng ml^−1^).

### Analysis of outcome

Progression-free survival (PFS) was calculated for each patient from the date of registration to the date of documented tumour progression, or death. Patients who discontinued the treatment early because of toxicity, refusal, or reason other than progression, were considered as censored at that time interval. Overall survival (OS) time was calculated for all patients from the date of registration to the date of death for any cause, or to patients last follow-up. Survival curves were generated by actuarial method (Kaplan and Meier, 1958), and compared by the log-rank test (Peto *et al*, 1977).

A logistic regression analysis was performed to identify the interaction between probability of response and pretreatment characteristics, while the Cox multivariate analysis (Cox, 1972) was carried out to assess their effect on time to progression and survival.

## RESULTS

### Patients and treatment

Patient main characteristics are listed in Table 1

**Table 1:** Main patient characteristics according to age grouping

	**Age groups (years)**			
**Characteristics**	**⩽54 *n*=37**	**55–69 *n*=64**	**⩾70 *n*=17**	**Total *N*=118**
Median age in years (range)	48 (28–54)	64 (55–69)	68 (65–79)	62 (28–79)
Males	14	41	14	69
Females	23	23	3	49

*Primary*				
Colon or recto–sigmoid	27	48	13	88
Rectum	10	16	4	30

*Previous surgery*				
No	5	11	1	17
Yes	32	53	16	101
*Previous adjuvant CT*				
No	26	50	13	89
Yes	11	14	4	29

*Synchronous metastasis*				
No	18	28	9	55
Yes	19	36	8	63

*Performance status*				
0	20	38	12	70
1	15	24	5	44
2	2	2	0	4

*Presence of symptoms*				
No	20	43	13	76
Yes	17	21	4	42

*Weight loss ⩾5*%				
No	28	53	12	93
Yes	9	11	5	25

*No. disease sites*				
1	20	36	10	66
>1	17	28	7	52

*Liver involvement*				
No	12	14	8	34
Yes	25	50	9	84
Single	4	12	0	16
<25%	12	23	4	39
>25%	9	15	5	29

*Lung involvement*				
No	31	49	13	93
Yes	6	15	4	25

*CEA basal value* ⩾100				
No	24	42	11	85a
Yes	11	22	6	20a

^a^No available basal values in 13 patients.

, according to age grouping. An imbalance in gender distribution was observed, given that elderly patients were more likely to be male (*P*=0.004). Other baseline characteristics were well matched across the three groups.

Younger patients stayed somewhat longer on treatment, while a similar length of treatment (and number of cycles) was administered to middle-aged or elderly patients (Table 2

**Table 2:** Duration of treatment, number of delivered cycles, and reason for discontinuation of therapy according to age grouping

**Age groups (years)**	**⩽54 *n*=37**	**55–69 *n*=64**	**⩾70 *n*=17**	
Stay on treatment (weeks)				
Median	24	15	14	*P*^a^=0.091
Range	2–42	2–45	2–35	
*Administered cycles*				
Median	9	7	7	*P*^a^=0.045
Range	2–16	1–20	1–12	

*Reasons for treatment discontinuation:*				
As for protocol	32	40	13	
Toxicity	0	7	0	
Refusal	1	4	4	*P*^b^=0.016
Disease complications	1	5	0	
Death	0	5	0	
Physician's decision	3	3	0	

a Kruskal–Wallis test.

b Pearson's *χ*^2^ test.

). Analysis of reasons for treatment discontinuation revealed that younger patients were less likely to refuse chemotherapy as compared to the middle-aged or elderly ones (*P*=0.016), while other causes were evenly distributed among the three groups.

A greater (although not statistically significant) proportion of elderly patients (35%) had a dose reduction along the first four cycles, in comparison with the middle-aged (25%), and younger patients (19%). In addition, a >2-week delay during the administration of the first four cycles was applied in 40% of elderly, in 25% of middle-aged, and in 26% of younger patients. During the four subsequent cycles, such a delay was applied in 62% of elderly, in 64% of middle-aged, and in 65% of younger patients. As a consequence, the median DI_4_ of cytotoxic drugs was slightly lower for elderly patients, but DI_8_ was substantially comparable in all groups (Table 3

**Table 3:** Absolute dose intensity (mg m^−2^ week^−1^) over the first four (DI4) and eight (DI_8_) cycles according to age grouping

	**Age–groups (years)**			
**Dose intensity**	**⩽54 *n*=37**	**55–69 *n*=64**	**⩾70 *n*=17**	***P*^a^**
DI_4_				
IRI				
Median	86	82	66	0.230
Range	55–100	52–100	41–100	

5FU				
Median	366	361	270	0.131
Range	231–425	249–458	175–426	


DI_8_				
IRI				
Median	85	75	81	0.532
Range	58–98	50–100	50–100	

5FU				
Median	366	322	345	0.449
Range	230–410	230–41	209–425	

a Kruskal–Wallis test.

).

After discontinuation of study treatment, 55 patients (47% of the whole series) received second-line therapy: 19 (51%) younger patients, 28 (44%) middle-aged patients, and 7 (41%) elderly patients (*χ*^2^ for trend not significant).

### Safety

Main severe haematological toxicity of this regimen was neutropenia, which occurred in 46 patients on the whole: grade ⩾3 neutropenia was detected in 43% of younger, in 41% of middle-aged, and in 31% of elderly group. Only four patients suffered from neutropenic fever or infection: their age ranged between 45 and 64 years. Among nonhaematological side effects, grade ⩾3 diarrhoea was reported in 11% of younger, 18% of aged, and 6% of elderly patients (*χ*^2^-test not significant). Severe stomatitis occurred in three patients on the whole: one patient in each age-group. Occurrence of other adverse events was not different in the three age groups (Table 4

**Table 4:** Severe (WHO grade ⩾3) toxicity according to age grouping

	**Age–groups (years)**		
**Toxicity WHO grade ⩾3**	**⩽54 *n*=37**	**55–69 *n*=64**	**⩾70 *n*=17**
Neutropenia	43	41	31
Anaemia	0	2	6
Thrombocytopenia	0	3	6
Neutropenic fever/infection	5	3	0
Diarrhoea	11	18	6
Stomatitis	3	2	6
Nausea/vomiting	5	0	6
Alopecia	51	36	12
Hepatic	0	2	0
Cholinergic syndrome	2	1	
Any type (except for alopecia)	68	70	50

Numbers are percent of patients.

). Interestingly, a lower proportion of elderly patients suffered from at least one episode of grade ⩾3 toxicity of any type (excluding alopecia) in comparison with aged or younger patients. However, seven patients, all belonging to the *middle-aged group*, went off-study for toxicity (diarrhoea, six cases; severe bone marrow suppression, one case). Treatment-induced or treatment-exacerbated early death (as defined by Rothemberg *et al*, 2001) occurred in three of 118 (2.5%) patients: a 62-year-old woman, completely asymptomatic at study entry), died of severe diarrhoea and dehydration after two courses; another woman, aged 63 years, and with initial PS=1, suffered from deep phlebitis with subsequent pulmonary embolism and acute cardiac failure after three cycles; and a 69-year-old man, entered into the study in good PS and no cardiac contraindications, had a fatal myocardial infarction after three cycles.

### Activity

As already reported, the IRIFAFU regimen produced nine complete plus 33 partial responses, for an overall response rate (RR) of 36% (95% confidence interval, 28–44%), according to intent-to-treat analysis. In addition, 12 patients showed a tumour shrinkage that did not qualify for a major response. RR was comparable in all age groups: it was 38% in the younger, 34% in the middle-aged, and 35% in the elderly patients. Similarly, no statistically significant difference in RR was observed according to gender (males, 36%; females, 35%); primary site (colon, 37%; rectum, 30%); previous adjuvant chemotherapy (yes, 41%; no, 34%); onset of metastasis (synchronous, 35%, metachronous, 36%); basal CEA serum level (<100 ng ml^−1^, 36%; ⩾100 ng ml^−1^, 40%). Some baseline characteristics, although not reaching a significant *P*-value, seemed affecting more the probability of response. This holds true for number of disease sites (single, 42%; multiple, 27%), performance status (0, 41%; ⩾1, 27%), disease symptoms (absent, 41%; present, 26%), previous loss of body weight (absent, 39%; present, 24%), previous radical surgery (yes, 38%; no, 23%). Keeping these last variables together with the age grouping into a logistic regression analysis, no significant interaction was reported with the activity rate.

### Long-term outcome

After a median follow-up of 30 months, 91 (77%) patients had progressed, and 75 (64%) had died. Besides the already mentioned three early deaths, two other patients died for a disease- and treatment-unrelated reason: a 66-year-old man committed suicide after 4 months from initial treatment, and a 36-year-old patient affected by insulin-dependent diabetes died of uncontrolled metabolic coma after 3 months from the start of therapy. Median PFS was 7.4, 8.0, and 5.3 months, for younger, middle-aged and elderly patients (log-rank test not significant also when adjusted by gender). In the multivariate analysis, taking into account all pretreatment characteristics, the presence of disease-related symptoms was the only variable significantly associated with a shorter PFS (*P*=0.053), while age group was of borderline significance (*P*=0.099). Survival, which at the Cox analysis resulted significantly affected by number of disease sites and previous loss of body weight, was unrelated to the age of patients: indeed, median OS was 13.4, 15.3, and 13.9 months, respectively, for the three age groups.

## DISCUSSION

In this paper, we have retrospectively reported on a series of patients affected by advanced colorectal carcinoma, randomly allocated to receive the IRIFAFU regimen, with the aim to ascertain whether the safety and activity of this regimen could have been affected by the age of patients. For this purpose, patients were divided in three age groups. Apart from sex, other baseline characteristics were evenly distributed across the three groups.

Considering the occurrence of treatment-related adverse events, as registered by the investigators, it appeared that severe toxicity was not greater in elderly as compared with other patients. An usually uncomplicated neutropenia affected a similar proportion of patients, regardless of age. Occurrence of severe diarrhoea was even lower among elderly patients. On the whole, a smaller proportion of elderly patients suffered from any type of severe toxicity. However, delays or dose reductions were applied during the initial treatment more frequently for elderly than for remaining patients. This apparent discrepancy may be explained by an earlier onset of side effects in elderly patients. Indeed, dose intensity over eight cycles was not different across the age groups, which is an indirect evidence that, with a cautious and tailored approach, our regimen was feasible also in elderly patients.

As for treatment discontinuation, it is worth noting that no young patient refused the study treatment, in contrast with four patients each in the two other groups. This observation may suggest that, despite toxicity was not more pronounced among these patients, the subjective perception of treatment-related physical and/or psychological distress was in these cases no longer tolerable. At this proposal, we would remember that a mental depression, which is a common psychiatric syndrome in elderly subjects, may be even more frequent in cancer patients. The presence and severity of this syndrome, when not sought and properly managed by the attending physicians, may adversely affect the compliance of patients to the anticancer treatment.

Although the results reported here were generated by a retrospective analysis, which was not powered to reveal significant differences according to age of patients, we believe meaningful that the proportion of responders was similar in all age groups. In addition, while a slightly poorer median PFS was registered for elderly, no difference at all in OS was noted for these patients as compared to middle-aged and younger ones. In this respect, a careful check for the presence of disease-related symptoms, or for a recent loss of body weight, together with an appropriate assessment of disease extent, showed a greater prognostic significance than the ECOG PS score.

Of course, this basically descriptive analysis cannot permit to draw firm conclusions, but it could help in generating hypotheses for future studies, in which the clinical benefit for elderly patients should be prospectively outweighed against the occurrence of side effects. Indeed, we have to admit that patients aged ⩾70 years were under-represented also in our trial. Although we may confirm that all eligible patients were offered to entry into this study, we cannot exclude a systematic selection bias, due to some reluctance of attending caregivers in referring elderly patients to cancer centres, or because of a poor willingness of patients themselves to take part in a randomised trial, or to undergo chemotherapy at all.

Moreover, in our case series no information was collected about the number and types of associated diseases, which may adversely affect the long-term outcome of patients (Extermann *et al*, 1998; Yancik *et al*, 1998). This was mainly due to the lack of a standard scoring system for co-morbidities in cancer patients (Repetto *et al*, 2002). Furthermore, a comprehensive geriatric assessment, including an evaluation of functional, cognitive and psychological status, has been strongly recommended for aged patients, in order to anticipate the acceptance and tolerability of a cytotoxic treatment (Balducci and Yates, 2000; Kearney *et al*, 2000; Köhne *et al*, 2001).

In conclusion, we suggest that geriatrics and medical oncologists should tightly cooperate in carefully evaluating elderly colon cancer patients, in order to identify those who may benefit from irinotecan plus FU combinations, and excluding patients with medical contraindications, or with an impairment of their physical, cognitive and/or psychological status. Furthermore, we strongly recommend the implementation of prospective trials to assess properly the quality of life of elderly patients undergoing chemotherapy. In this way, it could be possible to counteract an unjustified ‘ageism’, a prejudice that denies opportunities of treatment or even cure for patients that, as far as we know, may have the same chance as younger people (Potosky *et al*, 2002). Apart from the obvious ethical and juridical implications of such a negative attitude, we would remember that a recent economic evaluation of healthcare intervention has quantified an additional yearly cost of about $1200 for elderly cancer patients receiving specific treatments (Hayman *et al*, 2001). In our opinion, this extra expense is by far included in the costs that a developed country can effort to manage these individuals.
